# Precise application of water and fertilizer to crops: challenges and opportunities

**DOI:** 10.3389/fpls.2024.1444560

**Published:** 2024-12-06

**Authors:** Yingying Xing, Xiukang Wang

**Affiliations:** Key Laboratory of Applied Ecology of Loess Plateau, College of Life Science, Yan’an University, Yan’an, Shaanxi, China

**Keywords:** precision agriculture, water and fertilizer management, crop quality, soil regulation, intelligent equipment

## Abstract

Precision water and fertilizer application technologies have emerged as crucial innovations in sustainable agriculture, addressing the pressing need to enhance crop yield and quality while optimizing resource use and minimizing environmental impacts. This review systematically explores the latest advancements in precision water and fertilizer application technologies. It examines the integration of advanced sensors, remote sensing, and machine learning algorithms in precision agriculture, assessing their roles in optimizing irrigation and nutrient management. The study evaluates various precision techniques, including micro-irrigation systems, variable rate technology (VRT), and predictive modeling, along with their implementation in diverse agricultural settings. Furthermore, the review addresses the challenges posed by soil environmental heterogeneity and emphasizes the necessity for a scientific index system to guide precise applications. Advanced irrigation methods, such as subsurface drip irrigation and micro-sprinkling, improve water-use efficiency and reduce salinity levels, while precision fertilization techniques optimize nutrient uptake and minimize leaching. The integration of machine learning and remote sensing facilitates real-time monitoring and adaptive management, resulting in increased resource use efficiency and reduced environmental pollution. However, the effectiveness of these technologies is contingent upon addressing soil heterogeneity and developing standardized application indices. This review highlights the novel combination of advanced sensing technologies and data analytics in precision agriculture, enabling targeted interventions tailored to specific field conditions. It underscores the importance of integrating soil microbial community dynamics and biochemical indicators with precision management practices to enhance soil fertility and crop performance. Furthermore, the development of predictive models and time series analysis tools represents a significant advancement in anticipating and responding to changing environmental conditions. Precision water and fertilizer application technologies offer substantial benefits for sustainable agricultural practices by improving crop yields, enhancing resource efficiency, and mitigating environmental impacts. The strategic integration of these technologies with tailored agricultural practices and robust monitoring systems is essential for optimizing nutrient cycling and maintaining soil health. Addressing existing challenges through interdisciplinary research and collaborative efforts will further advance the implementation of precision agriculture, contributing to long-term soil sustainability and global food security.

## 1 Introduction

The advancement of agricultural science and technology, coupled with the urgent need for global food security, has brought the precise application of water and fertilizers to the forefront of sustainable agricultural development. Traditional farmland management often faces challenges such as excessive fertilization, inadequate irrigation, imbalanced water–fertilizer coupling, resource wastage, and environmental pollution (Chojnacka and Moustakas, 2024). Therefore, investigating the conditions for precise water and fertilizer application, as well as the mechanisms that enhance crop quality, increase yield, and regulate soil, is essential for promoting green agricultural development (Xing and Wang, 2024a).

Improving crop quality and yield involves optimizing nutrient supply and soil management to enhance crop performance while maintaining stable yields (Dhaliwal et al., 2022). Soil nutrient supply is a fundamental pillar of agricultural production, crucial for crop growth and development (Brown et al., 2022). However, traditional practices often lead to inefficient monitoring and application techniques, resulting in low fertilizer utilization, significant nutrient loss, and compromised crop quality and yield (Zhang et al., 2011). Thus, studying the technical conditions for precise water and fertilizer application is vital for achieving improvements in both crop quality and yield.

The mechanism of soil regulation encompasses the manipulation of the physical, chemical, and biological properties of soil to control nutrient supply and release, thereby enhancing soil fertility and crop yield. Understanding soil characteristics and nutrient transformation processes is crucial for precise nutrient regulation (Wei et al., 2024). By adjusting soil moisture content, aeration, and water-holding capacity, an optimal growth environment can be created that facilitates root development and nutrient uptake (Laghari et al., 2016). The incorporation of organic matter and microbial fertilizers can improve soil texture and structure, enhancing its capacity for water and fertilizer retention, ultimately increasing crop yield (Shu et al., 2022).

Research on precise water and fertilizer application conditions, alongside mechanisms that enhance crop quality, yield, and soil regulation, significantly promotes sustainable agricultural development (Peng et al., 2023). With global agriculture facing challenges such as limited land resources and water scarcity, improving the efficiency of water and fertilizer use and optimizing the soil ecological environment is a top priority (Wang, 2022). Furthermore, as consumer demand for high-quality agricultural products rises, the traditional model of prioritizing high yield and high fertilizer usage is becoming increasingly unsustainable (Yu et al., 2023).

## 2 Effects of combined application of water and fertilizer on crop quality

### 2.1 Analysis of crop growth characteristics

Optimizing irrigation practices is essential for maximizing banana production. Maintaining moderate soil water deficits promotes extensive root development and preserves fruit quality. The crop’s water demand varies significantly—from 1,200 to 2,690 mm per year—depending on the cultivar, developmental stage, and environmental conditions. Additionally, considering the unique ratooning cycle of bananas is crucial for maximizing water productivity and minimizing environmental impacts (Panigrahi et al., 2021).

Understanding crop growth cycles—which typically include germination, vegetative growth, flowering, fruiting, and maturity—is vital for effective agricultural management. Monitoring development across these stages provides valuable insights into growth patterns. During germination, evaluating germination rates and related indicators assesses a crop’s initial viability. Observations during vegetative growth help determine adaptability and growth vigor in response to environmental factors. In the flowering stage, tracking the development of floral organs and the duration of flowering allows for the measurement of key indicators such as flower bud count, flowering rate, and fruit set rate. Notably, despite claims of pollinator independence, the “Independence” almond variety exhibited a 60% increase in fruit set and a 20% enhancement in kernel yield when pollinated by bees, highlighting the critical role of pollinators even in self-fertile varieties (Sáez et al., 2020). Such detailed observations at various growth stages provide scientific evidence for precise water and fertilizer management aimed at improving crop quality and yield.

**Figure 1:**
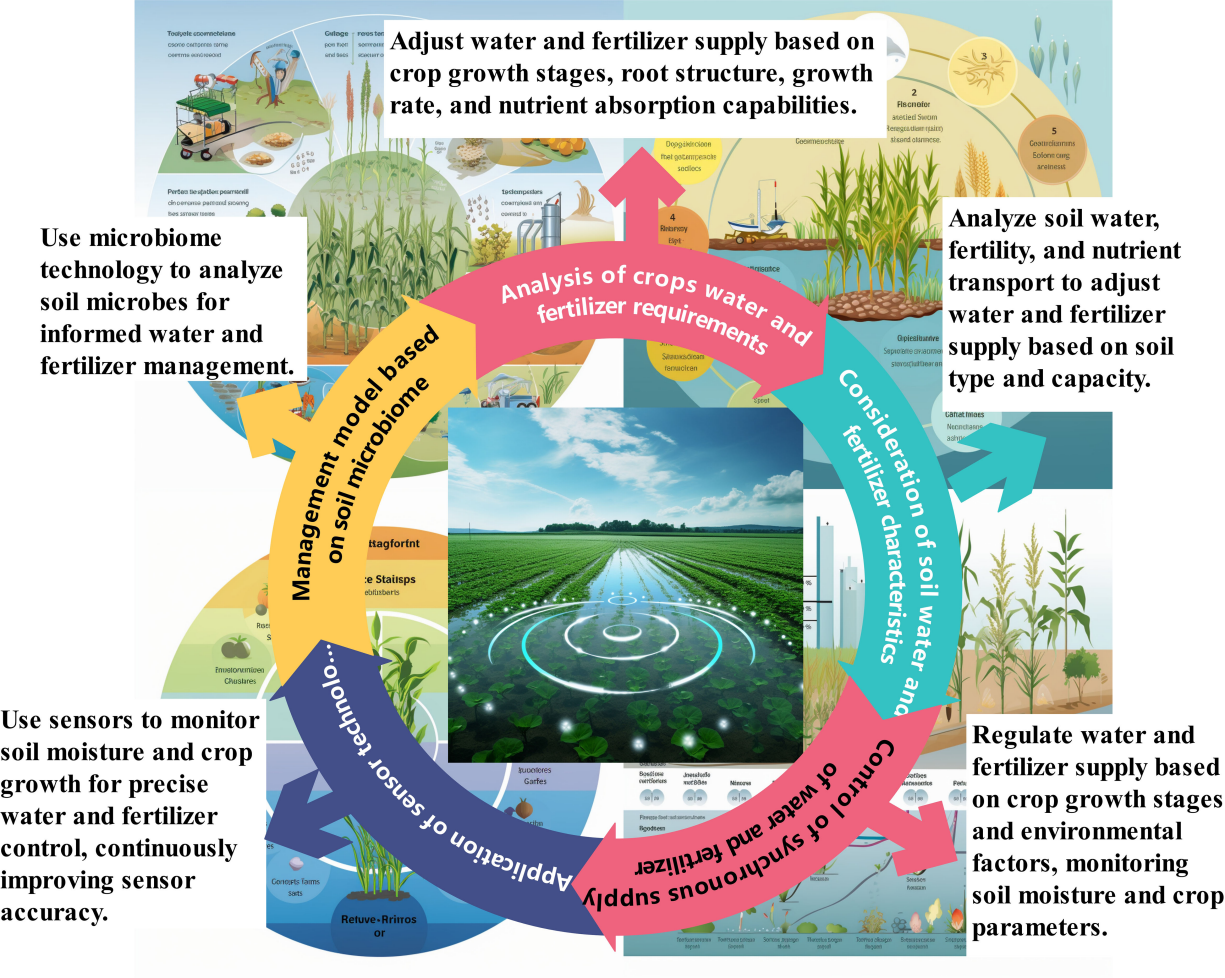
Mechanism of precise application of water and fertilizer on crop growth.

Crop biomass is a critical indicator for evaluating growth and potential yield. Accurate measurement of biomass offers insights into the status, growth rate, and total productivity of crops (Justes et al., 2021). Selecting optimal measurement methods tailored to specific circumstances ensures accurate assessments (Abbasi et al., 2020). Integrating biomass data with geographic and meteorological information enables comprehensive analyses of mechanisms to enhance crop quality and soil regulation (Liang et al., 2023). Continued research into the mechanisms and strategies of synchronized application will provide valuable insights and support sustainable agricultural development (**Figure 1**).

Accurate crop yield statistics are essential for assessing the effects of water and fertilizer management on agricultural output (Lobell et al., 2020). Given the variability in crops and treatments, statistical analyses are necessary to determine the significance of yield differences (Ye et al., 2020). Techniques such as variance analysis, regression analysis, and correlation analysis facilitate the identification of significant differences and the exploration of underlying mechanisms. Considering crop growth periods in statistical analyses is crucial, as yield patterns fluctuate throughout these phases (Wagg et al., 2021). Implementing standardized management practices during data collection ensures comparability and accuracy of results (Li et al., 2021b).

### 2.2 Evaluation of water and fertilizer application effects

Optimizing irrigation and fertilization strategies is crucial for enhancing crop yield, quality, and resource use efficiency. In the xerothermic regions of southwest China, Sun et al. (2022) identified an optimal schedule for mango cultivation that involves applying 75% of the crop evapotranspiration, along with sequential fertilization of 50, 75, and 25 kg ha^-^¹ at the flowering, fruit expansion, and fruit ripening stages, respectively. This strategy not only achieves high yield and quality but also significantly improves water use efficiency (WUE) and partial factor productivity (PFP) by 20% and 25%, respectively, compared to full irrigation.

Several factors influence WUE and nutrient use efficiency (NUE), including soil fertility, application methods, crop variety, cultivation management, and climatic conditions (Alkharabsheh et al., 2021). Implementing controlled-release urea at 150 kg N ha^-^¹ for rice and 120 kg N ha^-^¹ for other crops, in conjunction with common urea (30 kg N ha^-^¹) and straw incorporation, has been shown to enhance NUE and improve grain quality and soil fertility. Although this approach resulted in a yield reduction of up to 29.8% compared to local farming practices, it offers a sustainable compromise for agriculture in southern China (Liu et al., 2021a). The use of biofertilizers, such as phosphorus-solubilizing bacteria (PSBs), can further improve crop growth and soil nutrient availability. Inoculation with PSBs HRP2, SSP2, and JRP22 significantly enhanced the growth of Chinese fir, increasing plant height by 1.26-fold, stem diameter by 40.69%, and root, stem, and leaf biomass by 21.28%, 29.09%, and 20.78%, respectively (Chen et al., 2021a). These findings highlight the potential of PSBs as eco-friendly biofertilizers that enhance soil nutrient content and uptake.

Examining both traditional and precision management techniques is essential for assessing potential yield improvements. Fertilization methods affect nutrient supply and the crop growth environment, thereby influencing yield outcomes (Toksha et al., 2021). For instance, an optimal fertilization regime comprising 315 kg N ha^-^¹, 210 kg P_2_O_s_ ha^-^;¹, and 285 kg K_g_O ha^-^¹ resulted in a lettuce yield increase of up to 42.42% and improved quality traits. Notably, nitrate content was directly proportional to the nitrogen rate, while soluble sugar and vitamin C levels showed positive correlations with nitrogen and phosphorus applications, respectively (Hong et al., 2022). These findings underscore the significant impact of balanced nutrient management on crop performance. Innovative analytical methods contribute to agricultural efficiency by enabling precise monitoring of nutrient and contaminant levels. A novel technique employing a platinum-coated tungsten-coil atom trap for lead analysis demonstrated a 32.5-fold increase in sensitivity compared to graphite furnace atomic absorption spectroscopy, achieving a limit of quantification of 11.0 ng L^-^¹ and a precision of 2.3% relative standard deviation (Atasoy, 2023).

The System of Rice Intensification (SRI), when integrated with nutrient management practices such as integrated nutrient management (INM) or organic fertilization, consistently outperforms conventional methods. This approach led to grain yield increases of up to 43.8% and improved nutrient uptake (Thakur et al., 2020). Enhanced root growth and physiological functioning contributed to superior grain formation, yield, and nutritional content, including elevated levels of nitrogen (N), phosphorus (P), potassium (K), iron (Fe), manganese (Mn), copper (Cu), and zinc (Zn). Quality indicators such as grain moisture, sugar, and protein content can be assessed through chemical analysis and microscopy to evaluate crop quality under various management treatments (Padilla et al., 2020).

**Figure 2:**
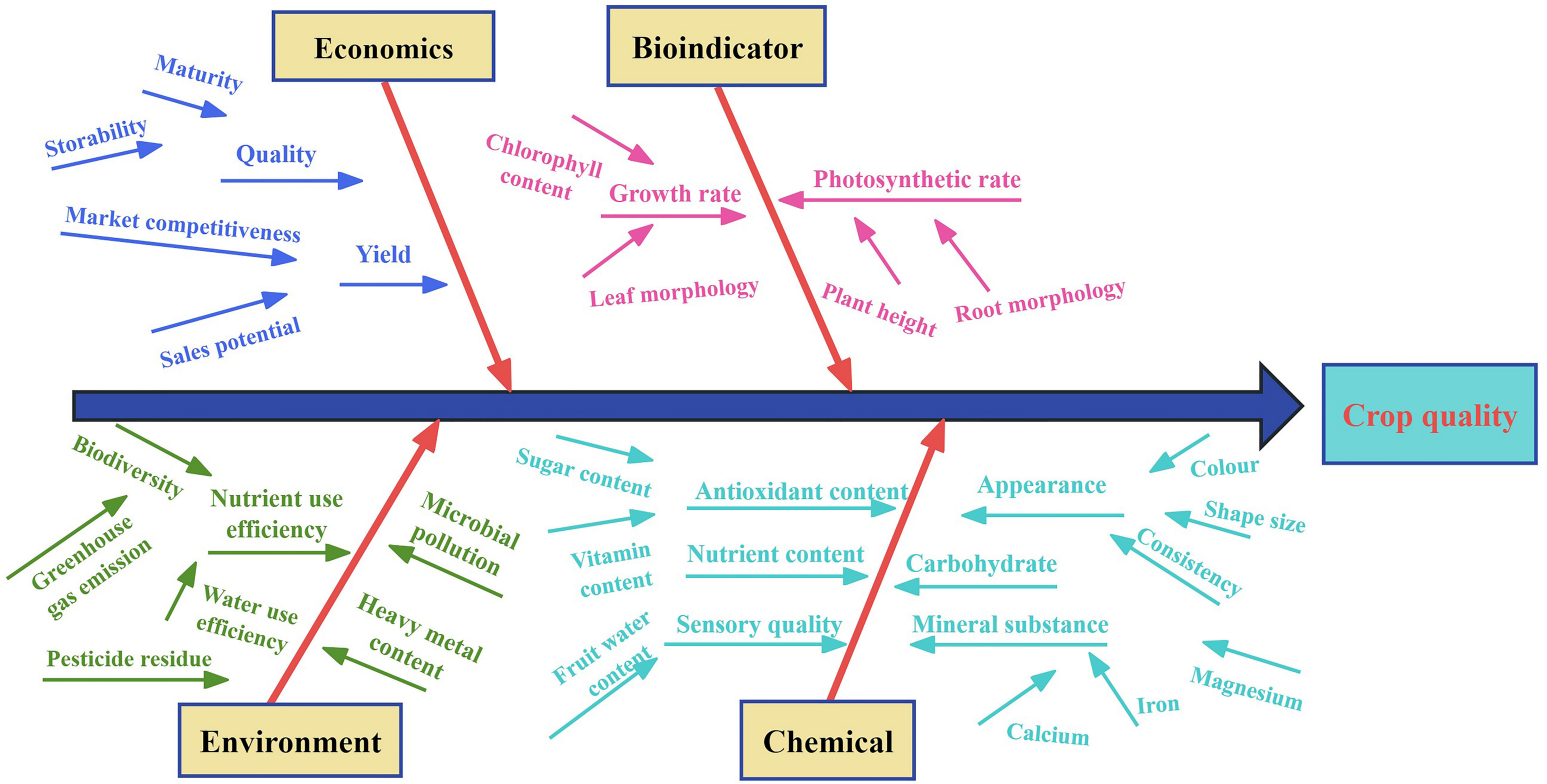
Crop quality evaluation index under precise combined application of water and fertilizer.

Effective phosphorus management is also critical for minimizing environmental impact. The application of spring-applied phosphorus fertilizer resulted in a 19% reduction in total phosphorus losses and a 33% reduction in dissolved reactive phosphorus (DRP) losses compared to fall broadcasting. However, the effects of cover crops on total phosphorus loss were inconsistent; they were associated with increased DRP loss in 75% of observed years (Carver et al., 2022). These findings emphasize the importance of phosphorus fertilizer management and highlight the mixed effects of cover crops on phosphorus loss mitigation, despite their consistent effectiveness in reducing sediment loss. In the context of precise water and fertilizer application, selecting appropriate evaluation indicators significantly influences the results (**Figure 2**). Innovations in analytical methods and a deeper understanding of nutrient dynamics further support sustainable agricultural practices.

### 2.3 Regularity of crop quality change

Quality indicators are essential for evaluating agricultural products, as they directly influence standards, market demand, and consumer preferences. Nutrient content, including proteins, fats, vitamins, and minerals, is critical. For instance, protein content serves as a key indicator of rice quality (Gaikwad et al., 2020), aligning with human nutritional requirements (Zhou et al., 2020). Appearance characteristics, such as color, shape, and size, significantly affect consumer purchasing decisions and market competitiveness (Chen et al., 2021b). Flavor quality, which encompasses taste, aroma, and texture, is vital for consumer satisfaction, particularly in fruits where sweetness, acidity, and texture are of paramount importance (Yun et al., 2022). The implementation of precise water and fertilizer applications can enhance these quality indicators, thereby increasing both market appeal and consumer satisfaction (Yan et al., 2024).

Under precise water and fertilizer management, various factors significantly influence crop quality. Systematic screening is necessary to identify key determinants. Soil characteristics, including nutrient content, pH, and texture, play a critical role (Milošević and Milošević, 2020). Investigating crop quality across different soil types can elucidate these influences. Additionally, climatic factors such as temperature, precipitation, and sunlight affect both growth and quality, underscoring the need to examine how these conditions impact crops. Furthermore, different crop types and varieties may respond variably to management practices, highlighting the importance of tailored approaches. For instance, a 50% reduction in urea and phosphate fertilizers in Region 3, identified as the primary source of pollution, resulted in an 18.1% decrease in nitrate leaching and an 8.35% reduction in phosphate leaching into the Aras River (Badrzadeh et al., 2022).

**Figure 3:**
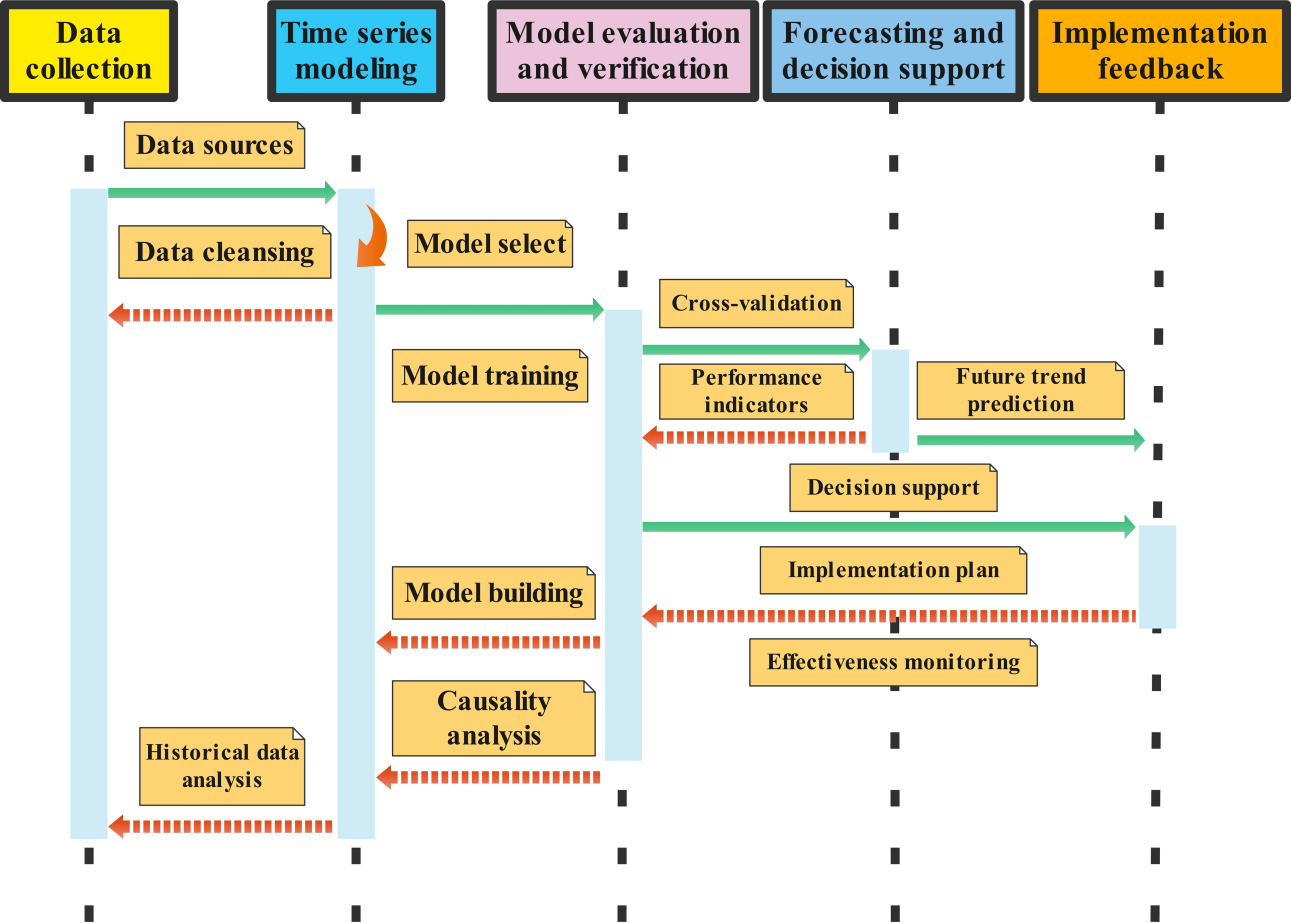
Application of time series analysis in the study of crop quality and soil control mechanism under the condition of precise combination of water and fertilizer. Data sources collect historical data on crop quality, including but not limited to growing environment data (e.g., temperature, rainfall, and light intensity), soil data (moisture, nutrient content), field management data (fertilization, irrigation records), and crop quality data (e.g., sugar content, protein content, yield, etc.). Data cleaning deals with missing values and outliers, standardizing data formats to ensure data accuracy and consistency. Historical data analysis (Growth stage) determines the changes in water and fertilizer requirements of crops at different growth stages and then optimizes the timing of fertilization and irrigation; (Climate impact) Analysis of crop quality changes under different climatic conditions, so as to adjust the water and fertilizer distribution strategy under specific climatic conditions. Model selection selects appropriate time series models according to data characteristics, such as the ARIMA model (autoregressive integral moving average model), the SARIMA model (seasonal ARIMA), and LSTM (long short-term memory neural network). Model training uses historical data to train models and adjust model parameters to improve prediction accuracy. In Model building, ARIMA (autoregressive integral moving average model), SARIMA (seasonal ARIMA model), LSTM (long short-term memory network), and other models were used for prediction and analysis. Causality analysis uses Granger causality test and other methods to determine the causal relationship between water and fertilizer distribution and crop quality change. In Cross-validation, the predictive power of the model is evaluated by cross-validation methods to avoid overfitting. In Performance indicators, mean square error (MSE), root mean square error (RMSE), and other indicators were used to evaluate the model performance. Future trend prediction uses trained models to predict trends in crop quality over a period of time. Decision support adjusts farmland management measures based on the forecast results, such as optimizing water and fertilizer application schemes to prevent possible quality degradation in advance. For Implementation plan, according to the forecast results and optimization plan, it is actually applied to farmland management. Effectiveness monitoring continuously monitors crop quality and yield, collects feedback data, and further optimizes models and management measures.

Time series analysis is a powerful method for examining and modeling data over time (**Figure 3**), facilitating the identification of trends and patterns that are essential for optimizing crop management (Aphalo and Sadras, 2022). A bibliometric analysis of 290 articles published between 1991 and 2022 revealed a significant positive correlation (*R*² = 0.937) between annual citations and the number of publications, indicating a growing interest in soil nitrogen losses associated with drip irrigation (Wei et al., 2024). Time series models can predict future trends in crop quality, enabling adjustments in water and fertilizer applications based on anticipated changes. For example, spring-applied phosphorus fertilizer resulted in a 19% reduction in total phosphorus losses and a 33% reduction in DRP losses compared to fall broadcasting (Carver et al., 2022). Conversely, the effect of cover crops on total phosphorus loss was inconsistent, with an increase in DRP loss observed in 75% of the years analyzed.

Climate change factors, including a temperature increase of 1.2°C and a water depletion of approximately 29 mm m^-^², have counteracted the 7% yield increase associated with rising CO_2_ levels (approximately 98 μmol mol^-^¹) in major wheat-producing countries over the past six decades. Notably, Germany and France have experienced net yield losses of 3.1%, while yields in China and the former Soviet Union are 5.5% lower than anticipated (Helman and Bonfil, 2022). These trends underscore the significance of predictive models and time series analyses in adapting agricultural practices to changing environmental conditions. Advanced modeling techniques, such as artificial neural networks, provide precise predictions for agricultural management. A developed neural network model accurately predicted sodium hazard levels in groundwater for irrigation in the Pratapgarh district of Southern Rajasthan, achieving a correlation coefficient of 1 and a root mean square error (RMSE) of 0.14 (Gautam et al., 2023). This model enabled the comparison of various nitrogen, phosphorus, and potassium ratios to identify the most effective application strategy. In conclusion, time series analysis and precise management practices are crucial for studying crop quality improvement and soil regulation. They facilitate the identification of patterns, optimization of application schemes, and prediction of future quality trends, providing valuable guidance for enhancing crop quality and yield efficiency (Kang et al., 2021).

### 2.4 Key factors affecting crop quality

Water and fertilizer management critically determines crop quality by directly influencing nutrient supply (Han et al., 2021). An optimal combination of irrigation (653.7 m³ ha^-^¹), nitrogen (1,141.9 kg ha^-^¹), and magnesium (422.1 kg ha^-^¹) fertilizers resulted in a cucumber yield of 88,412.6 kg ha^-^¹, increasing reducing sugars by 19.0% and free amino acids by 9.8%, while enhancing nitrogen use efficiency (Li et al., 2023b). The integration of organic and chemical fertilizers, especially with 60% cattle manure, significantly enhanced soil enzymatic activities and increased microbial biomass carbon and nitrogen by up to 62.2% and 54.5%, respectively (Iqbal et al., 2022). This approach yielded higher rice production compared to the exclusive use of chemical fertilizers, demonstrating a sustainable strategy for enhancing soil functionality and crop productivity.

Effective water management is vital for healthy crop growth and has a significant impact on overall quality (Li et al., 2021a). By optimizing irrigation practices according to growth stages and soil moisture levels, losses can be minimized, and efficiency can be improved, ultimately enhancing crop quality (Zhang et al., 2021a). Amending soil with a combination of biochar and straw has been shown to significantly enhance soil aggregate stability, achieving a Granular Stability Index of up to 98.74, while also improving water-holding capacity (field capacity, FC = 0.317 cm³ cm^-^³). Furthermore, this amendment increases dissolved organic carbon content to 272.828 mg kg^-^¹ and stabilizes air permeability under freeze–thaw cycles (Xue et al., 2022). Adjusting soil pH, organic matter, and trace elements within a combined management framework further promotes crop quality (Niu et al., 2021).

Maintaining optimal soil pH levels is crucial for enhancing crop quality, which includes improved vitamin content and increased disease resistance (Tripathi et al., 2022). A suitable pH establishes a favorable growth environment, facilitating better nutrient and water absorption (Antonangelo et al., 2021). Additionally, climate factors such as temperature, sunlight, and humidity play critical roles in crop growth (Malhi et al., 2021). For example, peanuts exhibit improved fruit color and taste at elevated temperatures, while tomatoes achieve higher nutritional content with adequate sunlight (Waraich et al., 2011). Therefore, regulating climate conditions is essential for enhancing crop quality. Appropriate levels of trace elements can also enhance plant metabolism and disease resistance (Kaur et al., 2023). The addition of selenium to soil improves wheat quality by increasing antioxidant levels and enhancing dough fermentation (He et al., 2023).

Analyzing the interaction between water and fertilizer under precise application conditions is essential for understanding improvements in crop quality and soil regulation. An adequate water supply maintains soil moisture, enhances nutrient absorption, and increases nutrient availability (Brouder and Volenec, 2008). Appropriate fertilizer application, including nitrogen, phosphorus, and potassium, provides essential nutrients that promote growth and quality (Yahaya et al., 2023). Key factors influencing these interactions include soil type, climate conditions, crop variety, and fertilization levels (Wang et al., 2019b). Climate factors such as rainfall and temperature also influence the decomposition and absorption of water and fertilizers (Maharajan et al., 2021). By simulating crop growth and nutrient processes, we can predict how these interactions affect crop quality (López-Pérez et al., 2018). A thorough understanding of these dynamics is essential for optimizing water and fertilizer management to enhance crop quality.

The interactive effects between water and fertilizer represent a critical mechanism influencing crop quality improvement and soil regulation under precise application conditions. Understanding these effects and the factors that influence them is vital for optimizing application schemes, enhancing crop quality, and efficiently utilizing soil nutrients. Consequently, future research should further investigate the mechanisms underlying water and fertilizer interactions to effectively improve crop quality and soil management.

## 3 Effects of physical and chemical properties of soil

### 3.1 Soil structure changes

Changes in soil structure play a crucial role in enhancing crop quality and optimizing soil conditions under precise water and fertilizer management. Applying organic amendments, particularly compost at 10 t ha^-^¹, significantly improves soil structure by enhancing aggregate stability, evidenced by a reduction in packing density (PAD) to 55% (Dong et al., 2022). Porosity also increases, with air-filled porosity reaching 0.29 cm³ cm^-^³ and soil organic matter (SOM) levels rising to 24 g kg^-^¹, contributing to enhanced hydraulic conductivity and gas diffusivity. These improvements promote soil health and facilitate increased crop growth.

Precise management mitigates soil compaction (Rivier et al., 2022) and improves soil structure, thereby enhancing crop quality and yield compared to traditional agricultural practices (Lin et al., 2024). Traditional practices often lead to uneven fertilization and excessive water use, resulting in nutrient leaching, compaction, and soil degradation (Huang and Hartemink, 2020). In contrast, precise management tailors nutrient and water provision to the specific requirements of the soil, preventing waste and avoiding salinization and acidification, which further enhances soil structure (Hopmans et al., 2021).

Precise water and fertilizer management increases SOM content and enhances soil structure stability (Cen et al., 2021). Excessive use of chemical fertilizers in traditional agriculture, such as NPK, can reduce soil organic carbon by 14% and destabilize soil structure. Conversely, organic amendments like vermicompost can increase soil organic carbon by 16% and improve soil microstructure, enhancing overall soil functionality (Rivera-Uria et al., 2024). The integration of 20 t ha^-^¹ of bio-organic fertilizer, coupled with a 20% reduction in chemical fertilizer, resulted in a decrease in soil bulk density by up to 0.22 g cm^-^³, an increase in total porosity by up to 8.30%, and an optimal cucumber yield of 23.45 t ha^-^¹ (Chen et al., 2022a). This demonstrates that the strategic use of organic amendments significantly enhances both soil physical properties and crop productivity in continuous cropping systems.

Land degradation drivers such as nitrogen enrichment and vegetation loss significantly reduce SOM decomposition rates. This reduction is mediated by changes in the alpha and beta diversities of rare soil bacteria and fungi, accounting for 33% of the variance in decomposition rates (Wu et al., 2021), underscoring the critical role of these rare microbial taxa in maintaining soil ecosystem functions. Traditional practices have led to a decline in soil microbial diversity and abundance, undermining soil stability (Hartmann and Six, 2023). In contrast, precise management creates a more favorable environment for microorganisms by limiting the use of chemical fertilizers and promoting organic applications, thus enhancing soil structure (Bamdad et al., 2022).

**Figure 4:**
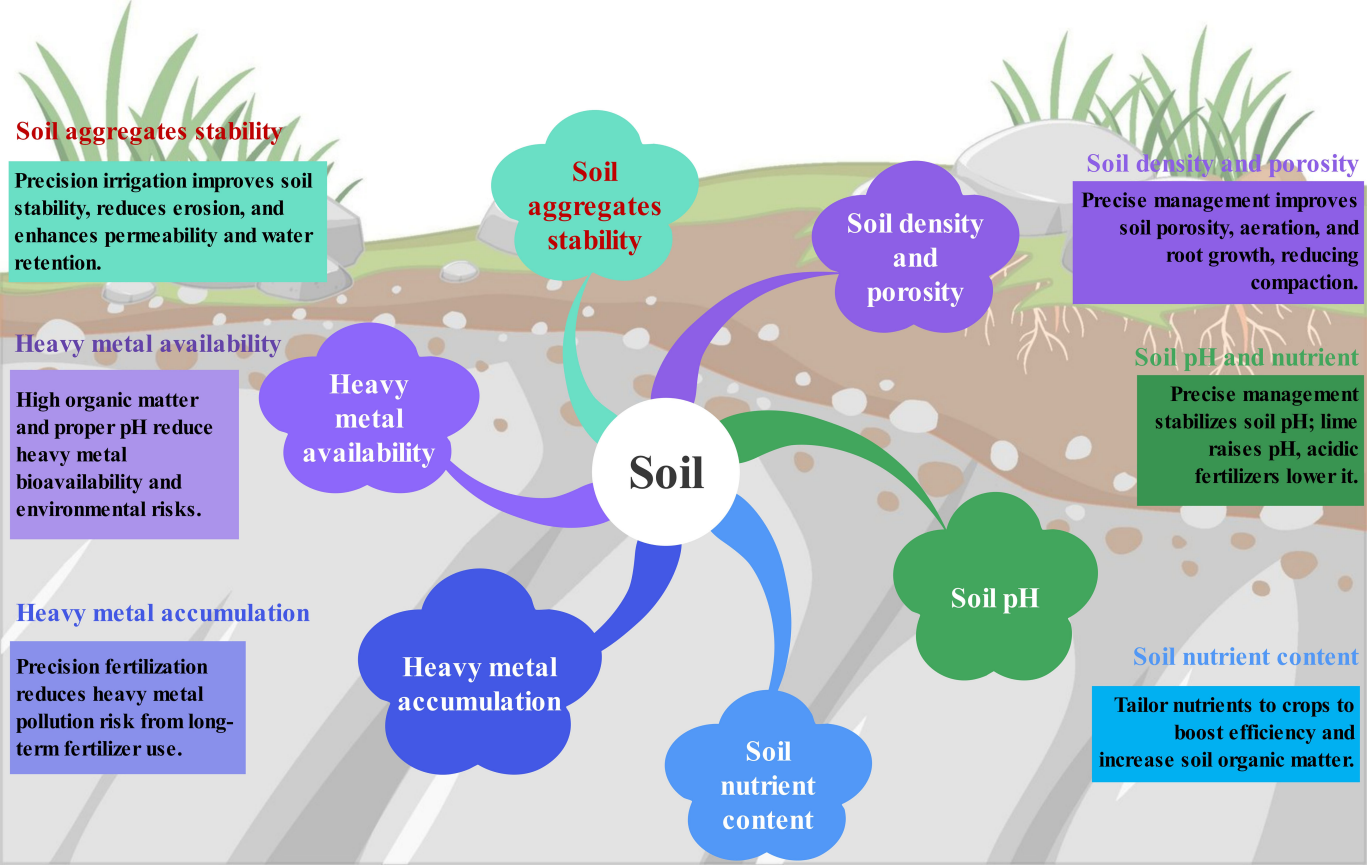
Response of soil structure, soil nutrients, and soil heavy metal content to precise application of water and fertilizer in farmland.

Effective water and fertilizer management is essential for enhancing soil structure, which, in turn, improves crop quality and yield. These practices reduce soil compaction, increase organic matter content, enhance microbial activity, and improve soil aeration, permeability, and water retention (Usharani et al., 2019). Collectively, these factors create a more favorable growing environment for crops, leading to increased yields and improved quality. Therefore, regulating soil structure through precise management is crucial for optimizing the soil environment and promoting sustainable agriculture (**Figure 4**).

### 3.2 Soil pH and nutrient dynamics

Soil pH has emerged as a primary factor influencing bacterial communities and their functions in agricultural soils. It exhibits significant positive correlations with both bacterial abundance and α-diversity (*p* < 0.05) and plays a crucial role in determining the distribution of key functional groups, including chemoheterotrophs (27.66% and 26.14%) and nitrifiers (6.87%) (Wang et al., 2019a). Changes in soil pH directly affect nutrient availability: low pH in acidic soils can increase the solubility of iron and manganese to potentially toxic levels, while high pH in alkaline soils reduces the availability of phosphorus, zinc, and copper (Barrow and Hartemink, 2023). Proper pH adjustments enhance nutrient uptake, thereby improving crop yield and quality (Briat et al., 2020). Acidic conditions inhibit beneficial microorganisms, thereby reducing nutrient supply (Dotaniya and Meena, 2015), whereas alkaline conditions adversely affect organic matter degradation and nutrient mineralization (Fatima et al., 2021). Adjusting soil pH fosters microbial activity and nutrient transformations, ultimately benefiting crop growth (Das et al., 2022). Additionally, low pH accelerates nutrient leaching (Ren et al., 2023), while high pH increases nutrient fixation, leading to deficiencies (Gu et al., 2023). Therefore, regulating soil pH is crucial for enhancing nutrient efficiency and overall soil fertility (Singh et al., 2021).

The application of poultry manure (5 t ha^-^¹) in conjunction with biochar (5 t ha^-^¹) resulted in significant yield increases for maize and black gram, with increases of 73% and 169%, respectively. This combination also led to a remarkable enhancement of net returns by up to 313%, underscoring the effectiveness of organic amendments in enhancing crop productivity and profitability within fully organic production systems (Das et al., 2024). Furthermore, organic amendments promote microbial activity and nutrient transformations, thereby improving soil fertility (Xu et al., 2021). Consequently, investigating the relationship between soil pH and nutrient dynamics is essential for optimizing water and fertilizer management, as well as for enhancing crop yield and quality.

### 3.3 Monitoring of soil heavy metal content

Monitoring soil heavy metal content is essential for understanding the regulatory mechanisms governing soil health. Heavy metal pollution has emerged as a significant environmental issue, adversely impacting farmland ecosystems and compromising crop safety (Qin et al., 2021). Consequently, accurate monitoring of heavy metals is crucial for safeguarding ecological environments and enhancing crop quality. The initial step in this process involves selecting appropriate monitoring methods, including chemical analysis, spectroscopy, and electrochemical analysis (Akhtar et al., 2021). Chemical analysis, the most widely employed method, entails soil sample collection, acid extraction, and atomic absorption spectroscopy to quantify heavy metal concentrations. Spectroscopy evaluates heavy metal content by exposing samples to specific wavelengths and measuring light absorption, scattering, and fluorescence. Electrochemical analysis identifies the presence of heavy metals by detecting variations in current.

Identifying target heavy metals is essential for monitoring soil contamination. Common targets include lead, cadmium, mercury, chromium, and zinc (Balali-Mood et al., 2021). Each metal possesses distinct toxicity and accumulation properties; therefore, the selection of targets should align with research objectives and the characteristics of the soil (Dhaliwal et al., 2020). Representative sampling points must be carefully chosen to prevent contamination during collection. Soil samples should undergo appropriate treatment, including the removal of impurities and drying, to ensure accurate results. Analyzing and interpreting monitoring data is critical, with statistical methods and geographic information systems aiding in the identification of temporal and spatial patterns as well as factors influencing soil heavy metal content.

Monitoring soil heavy metal content is a vital component of research aimed at enhancing crop quality and yield through precise water and fertilizer management, as well as effective soil regulation mechanisms. The careful selection of monitoring methods, identification of target metals, accurate sampling and processing, and rigorous scientific data analysis and interpretation provide a crucial scientific foundation for preventing soil heavy metal pollution and safeguarding the ecological integrity of farmland.

## 4 Effects of soil biological characteristics

### 4.1 Microbial community structure

The microbial community structure is a fundamental aspect of soil biology, encompassing the diversity, abundance, distribution, and interactions of microorganisms. Soil pH, which ranges from 3.47 to 7.47, serves as the primary edaphic factor influencing bacterial communities following mining disturbances (Xiao et al., 2021). It affects the availability of nutrients and metal(loid)s, significantly impacting microbial diversity, taxonomic composition, and functional gene enrichment. These changes, in turn, shape essential physiological, ecological, and geochemical soil processes. A 7:3 ratio of organic to chemical fertilizer optimally enhances the yield and quality of Euryales Semen by improving soil fertility, microbial diversity, and enzyme activity. This ratio accounts for 90.80% of the variance in both yield and metabolome, thereby establishing a strong foundation for the sustainable and high-yield cultivation of Euryales Semen (Li et al., 2023a).

Environmental factors such as soil pH, moisture, and temperature significantly influence microbial community structure (Fan et al., 2022). The development of glycerol-based media incorporating zinc ions effectively facilitates the isolation of acidophilic sulfate-reducing bacteria (aSRB) from acidic environments (Ňancucheo et al., 2016). This method enhances the detection and cultivation of aSRB by converting harmful hydrogen sulfide into insoluble zinc sulfide, thereby enabling colony differentiation based on metal sulfide deposition (Suzuki et al., 2021). Additionally, the type and concentration of organic matter significantly affect microbial composition, leading to the enrichment of various microbial taxa (Wang et al., 2021). Crop types and planting methods also modify the rhizosphere environment, consequently impacting microbial communities (Gong et al., 2023). The integration of cellulolytic nitrogen-fixing bacteria into lignocellulosic crop residue composting processes accelerates decomposition, enhances compost quality and nutrient content, and promotes sustainable agriculture by improving soil fertility and crop yields (Harindintwali et al., 2020). Conversely, nitrate-reducing bacteria are more prevalent in waterlogged crops such as rice (Lin et al., 2018). Furthermore, farmland management practices play a crucial role; the application of organic fertilizers enhances microbial diversity, while excessive use of chemical fertilizers and pesticides diminishes and alters microbial communities (Xing et al., 2024).

The structure of microbial communities critically influences soil biological characteristics and crop yield. In-depth research on the changes and regulatory mechanisms of microbial community structure under precise water and fertilizer management conditions can optimize farmland practices, promote sustainable agricultural development, and enhance both crop quality and yield.

### 4.2 Changes in microbial activity

Precise water and fertilizer management in farmland has led to significant changes in soil microbial activity. Microorganisms are essential to soil biology and play a crucial role in influencing soil health. This management approach notably enhances microbial populations by meeting the moisture and nutrient requirements of crops, thereby creating a more favorable environment for microorganisms (Liu et al., 2021b). Continuous cropping significantly disrupts the structure of soil microbial communities, leading to reduced crop yields and quality (Chen et al., 2022c). However, the application of synthetic microbiology, along with the optimization of living conditions, can effectively restore microbial balance and improve soil health.

The combination of biochar addition with daily fertigation significantly enhances soil quality and microbial activity, resulting in improved water and nutrient retention. This synergistic approach maximizes cucumber yield and water–fertilizer productivity in the alkaline soils of semi-arid regions (Zhang et al., 2020). This improvement leads to greater efficiency in microbial utilization of water and nutrients, more active metabolic processes, and enhanced physiological functions (Bargaz et al., 2018). Furthermore, this management approach promotes microbial diversity by stabilizing the soil environment, which creates more favorable conditions for various microorganisms. Consequently, microbial species both compete and cooperate, positively influencing soil functions (Philippot et al., 2024). Overall, precise management increases the number, activity, and diversity of soil microorganisms, thereby supporting improved crop yield and quality (Sabir et al., 2021). Investigating the mechanisms underlying these changes is essential for optimizing soil management strategies and enhancing the efficiency of water and fertilizer use in agricultural settings.

### 4.3 Enzyme activity impact

Low tillage frequency combined with the application of liquid dairy manure enhances soil organic carbon by up to 31% and nitrogen by up to 21%, and increases the activities of key enzymes such as N-acetyl-β-D-glucosaminidase (NAG) and phosphomonoesterase (PME). Conversely, it reduces the activities of phosphatase (PO) and protease (PP), indicating a shift in carbon cycling and improved nitrogen and phosphorus availability (Uwituze et al., 2022). Variations in enzyme activity reflect changes in microbial metabolism, organic matter decomposition, and nutrient availability (Xu et al., 2015). Enzyme activity is influenced by soil type, nutrient levels, and seasonal fluctuations. Different forest types across varying climates significantly impact soil microbial communities and enzyme activities, with soil moisture and organic matter content being critical factors. For instance, Ponderosa Pine and Mountain Hemlock forests experience extreme moisture and temperature conditions, resulting in specialized enzyme profiles (Brockett et al., 2012). Nutrient-rich soils enhance microbial activity, leading to increased enzyme activity (Zhang et al., 2022). In temperate hardwood forest soils, seasonal variations in temperature and moisture significantly enhance microbial and enzyme activity; approximately 63% to 69% of total annual enzyme activity occurs during the warm months, highlighting the critical role of climatic factors in organic matter decomposition and ecosystem functioning (Baldrian et al., 2013).

Enzyme activity plays a crucial role in crop growth by facilitating the decomposition of organic matter and the release of nutrients, supplying essential nutrition to plants (Fageria and Moreira, 2011). It also influences microbial metabolism, promoting the production of growth factors, including plant hormones (Mitra et al., 2022), which enhance disease resistance and stress tolerance (Govind et al., 2016). Therefore, regulating soil enzyme activity can improve soil conditions and support crop growth and development.

Regulating soil enzyme activity is essential for enhancing both crop yield and quality. Appropriate fertilization increases nutrient levels, promoting microbial and enzyme activity (Zhang et al., 2016). In subtropical paddy soils, combining organic and inorganic phosphorus sources is necessary to sustain soil microbial activity and minimize environmental impacts. Total phosphorus application should not exceed 44 kg P ha^-^¹ per year, as higher rates decrease acid phosphatase activity and pH levels (Zhang et al., 2015). Additionally, adjusting soil moisture and temperature can positively affect enzyme activity and microbial metabolism. By considering soil type, nutrient status, and environmental conditions, it is possible to effectively regulate soil enzyme activity to improve crop yield and quality.

Under precise water and fertilizer management conditions, investigating the impact of soil enzyme activity provides theoretical support for scientifically regulating soil environments and enhancing soil fertility. Effectively managing soil enzyme activity increases the soil’s nutrient supply capacity, improving crop growth and achieving the goals of precise water and fertilizer management. Therefore, in-depth research into the mechanisms and regulatory pathways of enzyme activity is essential for promoting sustainable agricultural development.

## 5 Soil–crop system interaction

### 5.1 Nutrient absorption and transformation

Nutrient absorption and transformation are critical processes within the soil–crop system. Nutrient absorption involves the active uptake of water-soluble inorganic ions by plant roots from the soil (Tianqian et al., 2024). This process is primarily facilitated by root activities, such as the production of root hairs, which expand the absorption area and enhance efficiency (Chen et al., 2022b). The growth of root hairs significantly influences the adsorption of nutrient ions in the soil (Ganther et al., 2022). Ion selectivity enables root hairs to preferentially absorb the most beneficial nutrient ions essential for plant growth and development (Meychik et al., 2021). However, factors such as root tip aging can affect nutrient absorption efficiency. Environmental contaminants can also impact root function. For instance, photoaged PVC microplastics significantly impair the root growth and architecture of wheat seedlings. Particles aged for 108 h reduce root length by 3.56% to 7.45% and induce substantial oxidative stress. This highlights the pronounced ecotoxicity of leached additives, such as Irgafos 168-ox and Irganox 1076, which warrant greater consideration in environmental impact assessments (Wang et al., 2024).

Nutrient transformation refers to the biochemical conversion of absorbed inorganic ions into organic substances, primarily occurring in the chloroplasts and cytoplasm of plants. Photosynthesis is a key process in this transformation, converting light energy into chemical energy through pigments located in the chloroplasts. This energy facilitates the synthesis of organic compounds from carbon dioxide and water (Stirbet et al., 2020). The products of photosynthesis, including glucose, sucrose, and starch, serve as essential nutrients for plant growth (Aluko et al., 2021). Additionally, plants convert inorganic nutrients into organic compounds such as amino acids, nucleic acids, and sulfate esters through the metabolism of nitrogen, phosphorus, and sulfur (Narayan et al., 2023).

Several factors influence nutrient absorption and transformation. An increase in soil nutrient content enhances a plant’s ability to absorb and convert these nutrients (Elbasiouny et al., 2022). Soil pH and temperature play crucial roles; optimal conditions facilitate root growth and nutrient uptake processes (Lyu et al., 2024). The dynamics of nutrients and water also influence the effectiveness of root traits in nutrient acquisition. For instance, in durum wheat, root angle traits affect phosphorus uptake. The “Narrow” genotype (41°) enhances deep soil exploration and phosphorus acquisition from deeper layers, whereas the “Wide” genotype (82°) is more effective in acquiring phosphorus from the topsoil (Van Der Bom et al., 2023). Understanding these interactions—which encompass root activities, ion selectivity for absorption, and the biochemical transformation of inorganic nutrients into organic substances—is essential for optimizing NUE (Li et al., 2024).

### 5.2 The relationship between soil and root growth

The relationship between root growth and soil is crucial for understanding the mechanisms of soil regulation. Roots play an essential role in enabling crops to absorb nutrients and water, which directly influences their development and yield (Gregory and Wojciechowski, 2020). However, soil conditions can constrain root morphology and function (Schneider and Lynch, 2020). Different soil textures significantly impact root growth. Clayey soils retain more water and nutrients but provide poor aeration, which can slow root development (Russo et al., 2020). The magnesium-based biochar-modified hydrogel slow-release fertilizer demonstrates a 47.12% increase in water absorption capacity, achieving a maximum of 1,395.12 ± 35.45 g g^-^¹. This notable enhancement in soil water retention and maize seedling growth offers a promising solution for sustainable agriculture in arid regions, as it diminishes irrigation needs and improves nutrient efficiency (Lang et al., 2023). Soil nutrients, particularly nitrogen, are vital for crop growth. Both insufficient and excessive nitrogen levels can adversely affect root development; the appropriate amount fosters growth, while an excess can inhibit it (Xu et al., 2023). Consequently, proper fertilization is essential for regulating nutrient supply and promoting root growth.

SOM plays a crucial role in enhancing soil structure, improving water and nutrient retention, and promoting root development (Xing and Wang, 2024b). Research indicates that increased organic matter content correlates with more robust and extensive root growth in crops (Sokol et al., 2019). Consequently, the application of organic fertilizers can effectively increase SOM and stimulate root development (Wei et al., 2016). Root hairs significantly enhance nutrient uptake and shoot growth in *Zea mays*, with their effects being more pronounced in loamy soils than in sandy soils. This finding underscores the critical roles of soil texture and root trait plasticity in nutrient acquisition and overall plant development (Vetterlein et al., 2022). Therefore, effective soil management requires scientific adjustments to these conditions to create an optimal growth environment for roots, ultimately enhancing crop quality and yield.

### 5.3 Water and crop growth interactions

Water is essential for crop growth and yield formation, with its interaction intricately linked to crop development. Effective regulation of water use is crucial for enhancing crop quality and yield, particularly under conditions of precise water and fertilizer application (Kang et al., 2017). Crop water requirements vary by growth stage, with increased demands during the seedling and flowering phases, while the demand decreases at maturity (Dietz et al., 2021). Timely water supply during these critical stages is vital for maximizing yields (Jin et al., 2020). In the soil–crop system, the supply of water is closely related to the water absorption capacity of roots (Zhang et al., 2023). Adequate water availability promotes root growth, enhances the absorption area, and supports nutrient transport (Cai and Ahmed, 2022). Proper water levels regulate crop temperature and create favorable conditions for photosynthesis and other physiological processes, thereby boosting growth and yield (Morales et al., 2020). Furthermore, water supply influences nutrient release and transformation in the soil, which, in turn, affects nutrient absorption efficiency (Zhang et al., 2021b). Sufficient water facilitates the transformation of nutrients such as nitrogen, phosphorus, and potassium, thereby enhancing their availability for crops (Sardans and Peñuelas, 2021). Conversely, inadequate or excessive water can impede nutrient release and conversion, diminishing their effectiveness and adversely affecting nutrient absorption (Palansooriya et al., 2023).

## 6 Opportunities and challenges of water and fertilizer precise application technology

### 6.1 Precision agriculture and sustainable soil management: enhancing efficiency and crop yields

Precision agriculture is a transformative approach that enhances resource efficiency and crop yields through advanced technologies. By integrating remote sensing, GPS guidance, and data analytics, farmers make informed, localized decisions. This optimizes inputs like water, fertilizers, and pesticides, reducing waste and environmental impact. Targeted interventions, such as variable rate application, address specific field needs, leading to increased yields and improved crop quality. Real-time monitoring of crop health and environmental conditions allows farmers to respond swiftly to changes, enhancing resilience to climate variability. Key technologies include remote sensing tools like satellite imagery and aerial photography, which provide detailed data on crop health, soil conditions, and environmental factors. Data analytics processes this information to identify patterns and optimize production practices. Leveraging these advancements, farmers manage operations more efficiently, sustainably, and profitably. Despite its benefits, implementing precision agriculture faces challenges. High initial costs for equipment and technology are significant barriers, especially in resource-constrained regions. Effective use requires technical expertise in data analysis, necessitating training and support. Managing and storing large data volumes poses difficulties, requiring robust data management strategies. Nevertheless, the long-term advantages—such as increased productivity, reduced environmental impact, and enhanced profitability—make it a valuable investment in sustainable farming.

Sustainable soil management is crucial for improving crop quality and yield through precise water and fertilizer application. Since soil is the foundation of crops, its sustainable use is essential. Strategies for sustainable soil utilization optimize water and fertilizer use, enhance yields, and protect the environment. Regulating soil physical properties through practices like deep plowing, loosening, and drainage improves soil structure and water and nutrient retention, and promotes root development. Regulating soil chemical properties is equally important. Rational fertilization, tailored to crop nutrient demands, prevents excesses and deficiencies, improving yield and quality. Fertilization plans that consider growth stages and balance nitrogen, phosphorus, and potassium are essential for optimal performance. Leveraging soil biological resources further enhances sustainability. Beneficial microorganisms and earthworms play vital roles in the soil ecosystem. Protecting and promoting soil biodiversity improves nutrient transformation, enhances crop resistance to pests and diseases, and reduces reliance on chemical pesticides, supporting sustainable agricultural ecosystems.

### 6.2 Integration and application of water and fertilizer precise application technology

Optimizing precision water and fertilizer application plans requires a thorough investigation and analysis of soil to evaluate nutrient status and crop requirements. Since different crops have unique nutrient needs, it is essential to scientifically determine the appropriate nutrient ratios based on crop characteristics and growth stages to meet these requirements and improve both yield and quality (Krouk and Kiba, 2020). Additionally, refining the application plan is vital for enhancing soil fertility and overall crop production capacity, which plays a significant role in promoting sustainable agricultural development and ensuring food security.

**Figure 5:**
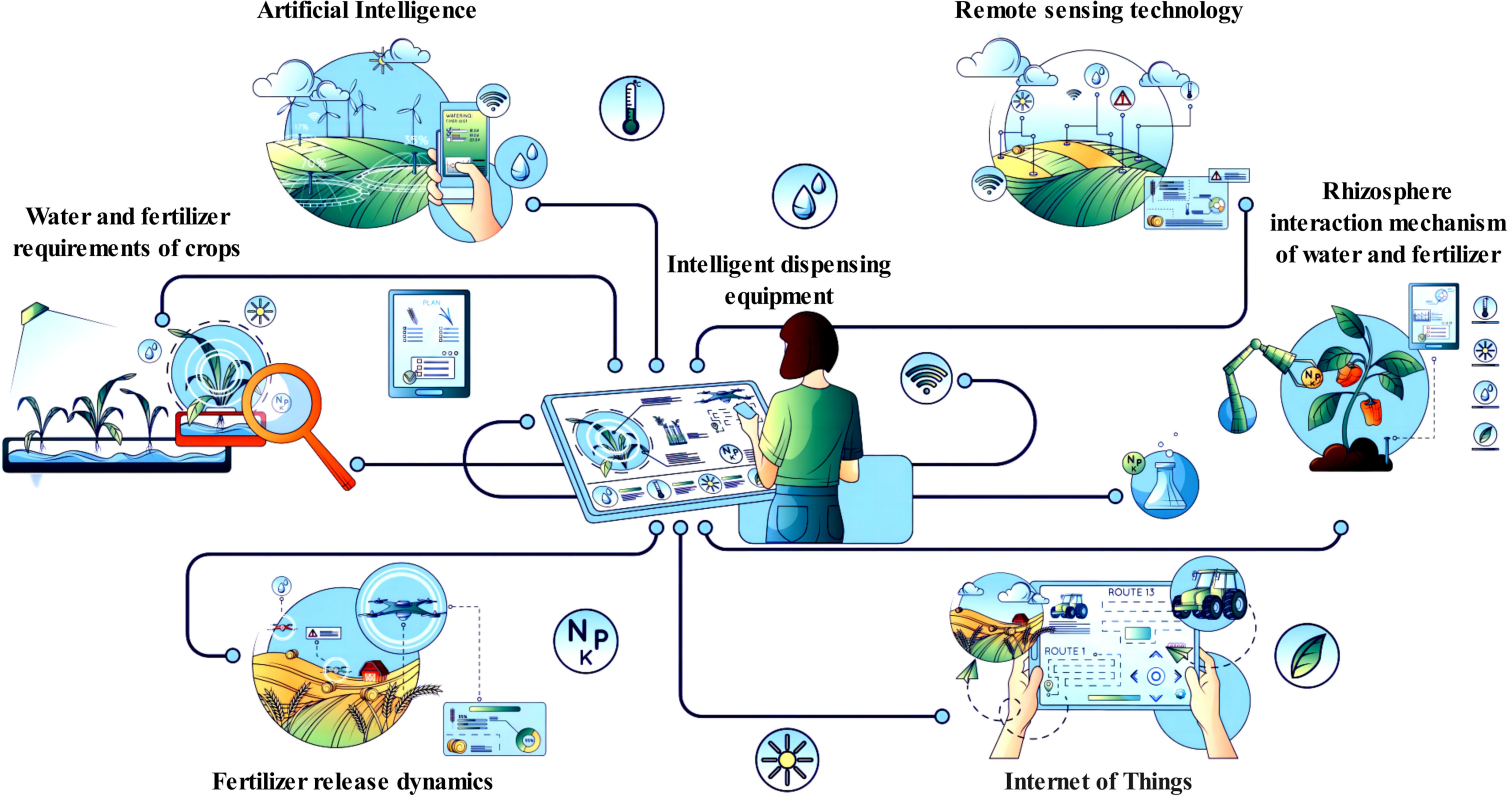
Through the intelligent distribution equipment assisted by the Internet of Things, remote sensing, and artificial intelligence, the law of crop water and fertilizer demand, the dynamic release of fertilizer, and the interaction mechanism of rhizosphere water and fertilizer are analyzed to implement precision agriculture. Intelligent dispensing equipment: automation equipment, control system, and feedback mechanism. Internet of Things: sensor, networked device, real-time monitoring, and data storage. Remote sensing technology: satellite remote sensing, UAV remote sensing, and ground remote sensing. Artificial Intelligence (AI): data processing and analysis, machine learning algorithm, prediction model, and decision support system. Water and fertilizer requirements of crops: water demand rule and fertilizer requirement rule. Fertilizer release dynamics: fertilizer type, release mechanism, and release model. Rhizosphere interaction mechanism of water and fertilizer: rhizosphere environment and interaction of water and fertilizer.

The design of control system integration is crucial for precision water and fertilizer application technologies. A comprehensive control system that leverages modern information technology is essential for the accurate application and regulation of water and fertilizers. The design of the control system integration should be based on modern information technology (**Figure 5**). Sensor technology, remote sensing, and geographic information systems can monitor soil moisture, soil fertility, and crop growth in real time, thereby providing vital data support for precision application. Furthermore, internet and Internet of Things technologies enable remote communication and data transmission, facilitating real-time monitoring and management by farmers.

The design of control system integration for precision water and fertilizer application technology must consider the specific conditions of the fields, including soil types, water and fertilizer management practices, and crop planting conditions. Furthermore, it should account for the operational capacity and economic feasibility for farmers. By leveraging modern information technology and integrating with environmental monitoring systems, while also addressing the actual conditions and needs of the fields, it is possible to develop efficient, user-friendly, and cost-effective control systems. These systems can optimize water and fertilizer usage, minimize waste and pollution, and contribute to the protection of the ecological environment, thereby supporting the sustainable development of agriculture.

Precision fertilization technology optimizes the use of water and fertilizers in agriculture, thereby enhancing production efficiency while minimizing pollution (Ladha et al., 2020). Analyzing soil nutrient content is essential for assessing fertility and developing a targeted fertilization plan. Determining the appropriate amounts of fertilizer based on crop needs and soil conditions ensures maximum efficiency. Furthermore, fertilization should align with the crop’s growth cycle, taking into account the varying nutrient requirements at different stages. In some instances, multiple applications or zone-specific methods may be necessary, tailored to the unique characteristics of both the crop and the soil.

### 6.3 Problems in the application of technology

Key issues in the application of precision water–fertilizer technology include low fertilization precision, challenges in integrating water and fertilizer, and the variability of crop nutrient requirements. The low precision often arises from traditional methods that rely on fixed ratios and timings, which inadequately address the diverse needs of various soils. Some soils may possess excess nutrients, while others may be deficient, underscoring the necessity for accurate soil nutrient assessment to enable effective integration. Although existing soil testing and rapid nutrient measurement techniques can enhance precision, further optimization remains essential.

The integration of water and fertilizer poses a challenge due to the varying requirements of crops for water and nutrients, complicating the determination of optimal ratios. Additionally, soil moisture conditions significantly impact this integration, as arid regions require more water than humid areas. Therefore, it is essential to establish water–fertilizer ratios that are tailored to specific soil and crop characteristics. While advancements in irrigation and fertilization technologies can improve the precision of this integration, further research and practical application are necessary.

The varying crop requirements for water and fertilizer present significant challenges in the application of precision water–fertilizer technology. Different crops exhibit unique nutrient needs; for instance, some require high nitrogen levels but low phosphorus, while others necessitate the opposite. Therefore, accurately determining the appropriate water–fertilizer ratios and timing based on these specific requirements is crucial for enhancing both yield and quality. Current research on crop growth and nutrient absorption can facilitate the optimization of this integration; however, further in-depth studies are essential.

**Figure 6:**
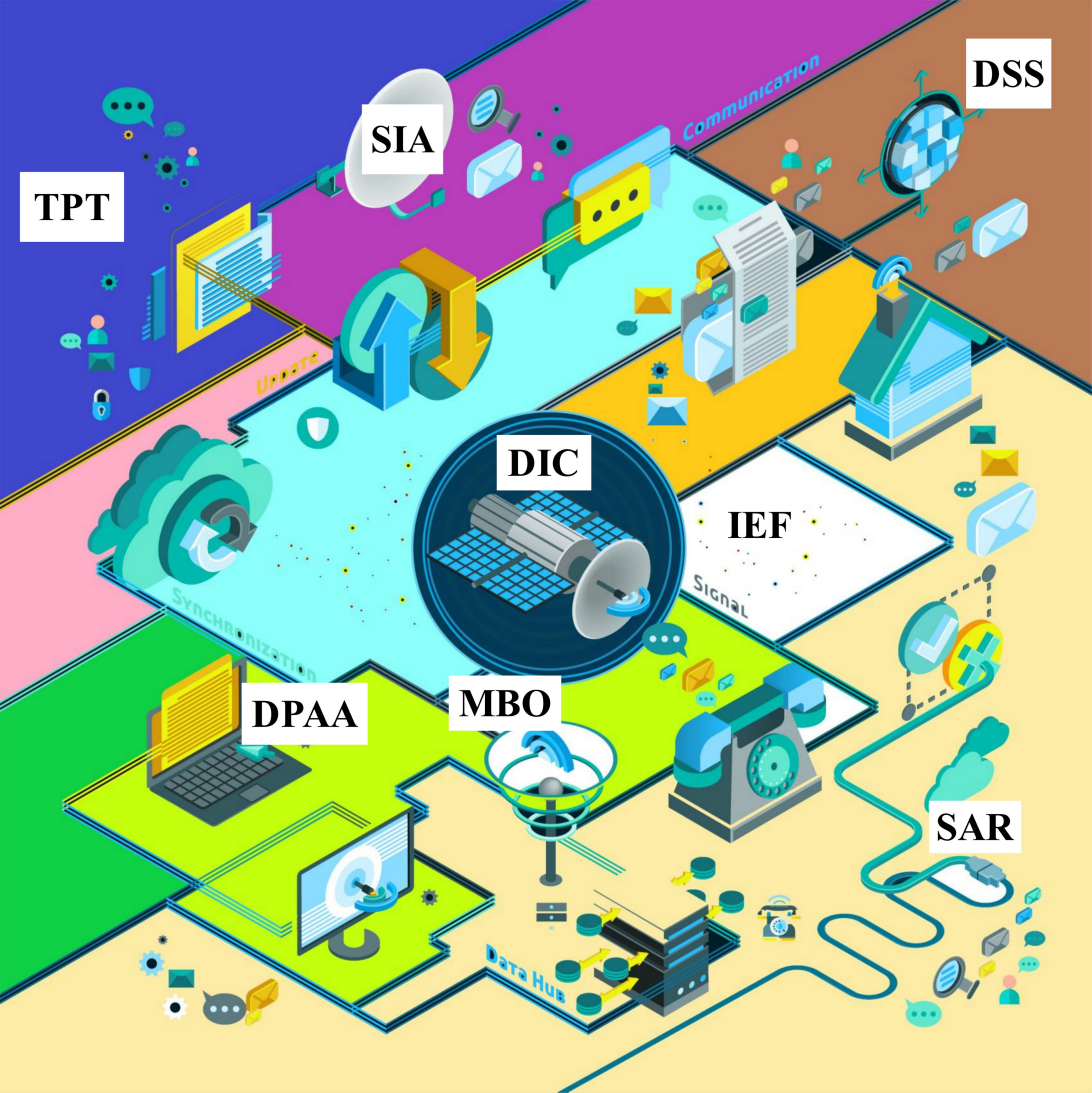
Intelligent distribution equipment to achieve accurate distribution of water and fertilizer need to solve scientific problems. SAR, sensor accuracy and reliability; DIC, data integration and consistency; DPAA, data processing and analysis algorithm; MBO, model building and optimization; IEF, impact of environmental factors; DSS, decision support system; SIA, system integration and application; TPT, technology popularization and training.

Addressing the issues of low fertilization precision, integration difficulties, and diverse crop requirements necessitates the refinement of precision technology, improvements in soil testing, and comprehensive research into crop demands. Optimizing fertilization methods and ratios is imperative (**Figure 6**). Only through scientific and technological advancements can effective water–fertilizer integration be achieved, ultimately improving crop yield and quality while ensuring sustainable agricultural development.

### 6.4 Analysis of environmental and economic benefits

Evaluating the environmental and economic benefits of precision water and fertilizer application technology is essential for its successful implementation in agriculture. This technology plays a significant role in promoting environmental protection and sustainable development. Firstly, it accurately assesses the water and fertilizer requirements of plants, thereby minimizing resource waste. Secondly, it helps reduce agricultural pollution; excessive fertilizer application can result in soil acidification and water contamination, but precision application effectively mitigates these issues, thereby safeguarding soil and water quality.

The application of precision water and fertilizer technology enhances agricultural production and promotes sustainable development. By accurately calculating the water and fertilizer requirements of plants, this technology minimizes resource waste, improves fertilizer efficiency, and reduces production costs. Furthermore, it decreases agricultural inputs and alleviates environmental pressure, thereby underscoring its economic benefits. Additionally, this technology improves soil quality and fosters crop growth by preventing nutrient accumulation or loss. It aids in regulating soil pH, enhancing soil structure, increasing organic matter content, and boosting water and nutrient retention, all of which collectively enhance soil fertility and create a more favorable environment for crops.

Precision water and fertilizer application technology provides substantial environmental benefits by reducing agricultural pollutant emissions, lowering greenhouse gas emissions, and mitigating climate change. Additionally, it decreases non-point source pollution, enhances water and soil quality, and safeguards the ecological environment. This technology not only improves environmental outcomes but also boosts economic performance by accurately determining the water and fertilizer requirements of plants, preventing over-application, and promoting resource efficiency while ensuring environmental protection. Economically, it reduces production costs and increases efficiency. Therefore, the adoption of this technology in agriculture presents promising prospects for future development.

### 6.5 Difficulties faced and countermeasures

Despite the significant achievements of precision water and fertilizer application technologies in enhancing crop yields and conserving resources, several challenges persist. A primary challenge is the establishment of a scientific index system for precise application, as controversies remain regarding the specific needs of different crops at various growth stages. Further research is necessary to develop a suitable index system that accommodates diverse farmland conditions and crop types, thereby improving both quality and production. Another challenge involves addressing the heterogeneity of soil environments, which is influenced by geographical factors, soil types, and texture. Effective application must account for this variability to adequately meet crop needs under differing conditions. Additionally, further study and optimization of soil improvement technologies are essential for enhancing soil quality and increasing the efficiency of fertilizer use by crops.

Implementing precision water and fertilizer technology requires a thorough understanding of various influencing factors, which presents a challenge for systematic research and evaluation. Current studies primarily rely on small-scale experiments, thereby limiting their applicability to broader agricultural contexts. To improve practical applications, it is essential to foster collaboration among scientists, farmers, and government entities to develop a systematic research and evaluation framework.

Precision water and fertilizer application technology is crucial for enhancing crop quality, increasing production, and managing soil conditions. However, challenges remain in its practical implementation. Addressing these challenges requires further research to establish a scientific index system, address soil environmental heterogeneity, and develop a systematic framework for research and evaluation. By undertaking these efforts, we can fully leverage the benefits of this technology, thus achieving sustainable farmland development alongside improved crop quality and yield.

### 6.6 Challenges in different agricultural regions

Water scarcity in arid regions presents significant challenges to agricultural productivity. Limited rainfall and high evaporation rates exacerbate this issue, highlighting the necessity for efficient irrigation practices to optimize water utilization. Advanced irrigation technologies, such as drip and subsurface irrigation, minimize water loss by delivering water directly to the root zone of crops. Additionally, moisture sensors enhance water efficiency by monitoring soil moisture levels and adjusting irrigation schedules accordingly. Furthermore, rainwater harvesting techniques and the cultivation of drought-tolerant crop varieties can play a crucial role in mitigating water stress. By implementing these strategies, arid regions can enhance agricultural sustainability and adapt to the challenges posed by water scarcity.

In humid regions, excessive rainfall poses several challenges, including waterlogging, nutrient leaching, and soil erosion. Waterlogged soils arise when saturation limits oxygen availability to plant roots, thereby hindering crop growth and potentially leading to reduced yields and increased susceptibility to diseases. Nutrient leaching is another significant concern, as heavy rainfall can wash away essential nutrients from the soil profile, depleting soil fertility and potentially contaminating water bodies. Furthermore, intense rainfall events can lead to substantial soil erosion, stripping away topsoil and degrading land productivity. To mitigate these challenges, farmers in humid regions can implement drainage systems to manage waterlogging, adopt cover cropping practices to prevent soil erosion, and utilize controlled irrigation methods to minimize nutrient leaching. By addressing these issues, agricultural productivity in humid regions can be sustained while also reducing environmental impacts.

Saline-alkali areas pose significant challenges to agricultural production due to the accumulation of salts and the poor structure of the soil. Elevated salt concentrations can hinder root growth and diminish the uptake of essential nutrients by plants, resulting in stunted growth and reduced yields. Furthermore, the suboptimal structure of saline-alkali soils, typically characterized by compaction and low organic matter content, further limits root penetration and water infiltration. These adverse conditions create a harsh environment for crop production, thereby constraining plant growth and productivity. To mitigate these challenges, farmers in saline-alkali regions can adopt strategies such as applying soil amendments to enhance soil structure, implementing leaching practices to decrease salt accumulation, and selecting salt-tolerant crop varieties. By addressing the specific limitations posed by saline-alkali soils, agricultural productivity can be improved, and sustainable land management practices can be established.

## 7 Summary and prospect

Precise fertilizer application enhances soil nutrient status and microbial activity, which subsequently improves crop yield and quality. Intelligent systems equipped with sensor technology facilitate real-time monitoring and regulation of water and fertilizer, thereby supporting these advancements. However, the review lacks a comprehensive analysis of the mechanisms underlying soil regulation. Future research should focus on soil microorganisms and nutrient cycling, employing molecular biology and ecological methods to investigate how precise applications influence microbial diversity and nutrient cycling. Additionally, advancements in implementation technology are crucial.

Future studies should incorporate advanced sensor technology and smart agricultural equipment to achieve more accurate and efficient management of water and fertilizer. Significant challenges persist in understanding the mechanisms that enhance crop quality and regulate soil health. Future research should prioritize experimental design, data collection, and the exploration of regulatory mechanisms involving soil microorganisms and nutrient cycling. The integration of relevant technologies will enhance and expand research efforts, contributing to increased yield, improved quality, and sustainable development in the precise application of agricultural water and fertilizer.
